# Spatial Associations and Co-Occurrence Networks of Sympatric Species in an Asian Elephant Community

**DOI:** 10.3390/ani16020351

**Published:** 2026-01-22

**Authors:** Jingshan Wang, Xu Li, Yuan Tian, Wenguan Duan, Yuhui Si, Dusu Wen, Weibin Wang, Dehuai Meng

**Affiliations:** 1 Key Laboratory of Conserving Wildlife with Small Populations in Yunnan, Key Laboratory of Forest Resources Conservation and Utilization in the Southwest Mountains of China, Ministry of Education, Southwest Forestry University, Kunming 650224, China; wangjingshan36@163.com (J.W.); lixu.swfu@hotmail.com (X.L.); syh3513@163.com (Y.S.); wendusu0227@126.com (D.W.); 2 Management and Protection Bureau of Yunnan Nangunhe National Nature Reserve, Lincang 677400, China; tiany0512@163.com (Y.T.); 18787494548@139.com (W.D.)

**Keywords:** *Elephas maximus*, ecosystem functioning, species interaction network, nature reserve, camera trapping

## Abstract

Understanding how species share resources (niche dynamics) and associate with each other is crucial for maintaining stable ecological communities. Using infrared camera traps, we constructed spatial association networks for an isolated Asian elephant population. The Asian elephant (*Elephas maximus*), a keystone species in tropical forests, faces significant threats from habitat fragmentation and human disturbances, particularly in the isolated population of Nangunhe National Nature Reserve, Yunnan, China. Using infrared camera trapping, niche analysis, and interspecific association models, we examined the ecological role of Asian elephants and their sympatric species networks in fragmented habitats. We identified 44 species, including 11 species with higher relative abundance showing significant ecological correlations with elephants. Asian elephants exhibited the broadest spatial distribution, consistent with their role as ecological engineers due to high environmental tolerance and diverse resource utilization. Sympatric herbivores exhibited moderate spatial co-occurrence. Wild boars (*Sus scrofa*), red-bellied squirrels (*Callosciurus erythraeus*), northern pig-tailed macaques (*Macaca leonina*), and red junglefowl (*Gallus gallus*) demonstrated significant spatial associations with elephants. Lambda coefficient analysis revealed asymmetric associations reflecting spatial reliance of red-bellied squirrels and wild boars on elephant activity zones. Temporally, Asian elephants exhibited a stable bimodal activity pattern at dawn and dusk. Despite varying degrees of diel overlap with sympatric species, no significant temporal avoidance was detected, suggesting fine-scale coexistence mechanisms beyond the temporal dimension. We argue that conservation strategies are in urgent need of a transformation from single-species protection to the preservation of ecological interaction networks. This study clarifies the dominant position of Asian elephants in the community by mapping the spatial association networks between Asian elephants and sympatric species, and its findings hold substantial guiding significance for the recovery and protection of isolated Asian elephant populations.

## Simple Summary

As a keystone species (a species that has a disproportionately large effect on its environment relative to its abundance), Asian elephants play a critical role as “ecosystem engineers” in their forest habitats. In this study, conducted in China’s Nangunhe National Nature Reserve, we explored how Asian elephants interact with other wildlife in a fragmented landscape. Our findings reveal that elephants have the broadest ecological influence, sharing their habitat with numerous other species. Animals such as wild boars and squirrels frequently live near elephant activity zones, possibly benefiting from the environmental changes elephants create. In contrast, species like macaques and junglefowl coexist more independently, occupying different parts of the habitat. This research demonstrates that protecting Asian elephants also supports a network of sympatric species, such as wild boars and red-bellied squirrels, which rely on elephant-modified habitats. This highlights the need to conserve not only individual species but also the web of ecological interactions. Our insights advocate for integrated conservation strategies that maintain these relationships, which will benefit both biodiversity and ecosystem resilience in fragmented landscapes.

## 1. Introduction

The Asian elephant (*Elephas maximus* Linnaeus, 1758), the largest terrestrial mammal in Asia, is classified as Endangered on the IUCN Red List and designated as a Category I National Key Protected Species in China [1]. Habitat fragmentation, human population growth, and escalating resource demands have led to the loss of more than 95% of its historical range. Currently, fragmented populations of Asian elephants persist across 13 countries in South and Southeast Asia, occupying a total habitat area of less than 500,000 km^2^ [2,3,4]. In China, Asian elephants are primarily distributed across the Xishuangbanna Dai Autonomous Prefecture, Lincang, and Puer in southwestern Yunnan Province. Rural population growth, agricultural expansion, and deforestation have disrupted migratory corridors, restricting elephants to fragmented habitat islands [5].

The Nangunhe National Nature Reserve (NGHNNR) in Lincang hosts a geographically and genetically isolated subpopulation of Asian elephants, with no gene flow recorded in populations within Myanmar for over a decade and genetic isolation from other Chinese populations for 50 years [6]. Currently, Asian elephants in Lincang are predominantly confined to three sectors, namely Banhong, Banlao, and Mangku, within the western core zone of the Cangyuan Section of the NGHNNR, with their actual activity range restricted to approximately 36 km^2^ [7]. As of 2023, the Asian elephant population in China is estimated at approximately 300 individuals [8]. Recent monitoring in the NGHNNR using infrared camera mark-recapture and microsatellite genotyping identified 17 and 19 individuals, respectively The limited habitat in the reserve may be insufficient to support the long-term population survival of the Asian elephant. Therefore, understanding the interspecific relationships within the Asian elephant community in this region is essential for the long-term survival and reproduction of the Asian elephant population.

As a keystone species, the Asian elephant critically shapes ecosystems through landscape modification, seed dispersal, and community structure regulation [9,10]. Climate and land-use changes pose ongoing threats to the remaining Asian elephant populations [3,11]. Human–elephant conflict has emerged as the most severe form of human–wildlife conflict in China, posing major challenges to both regional development and conservation efforts [12,13,14,15]. Effective conservation strategies must holistically consider the ecological roles of endangered species within community structures to avoid scenarios in which single-species conservation measures disproportionately affect sympatric species. Constructing robust species interaction networks is a critical technological approach for resolving ecological imbalances [16]. Interspecific associations qualitatively indicate the likelihood of species coexistence, whereas association coefficients between species pairs quantitatively reflect the strength of interspecific relationships [17,18]. Interspecific associations are generally shaped by community habitat characteristics and interspecific trait variations [19]. A balanced mixture of moderate antagonistic and mutualistic interactions can stabilize population dynamics, enhance community complexity, and increase overall ecosystem stability [20,21]. Exclusive focus on individual biotic assemblages overlooks their inherent dependence on interspecies interaction networks for long-term viability [22]. Consequently, an exclusive emphasis on species diversity preservation may fail to safeguard critical ecological network architectures; however, maintaining these interaction networks is crucial for biodiversity conservation [23].

Establishing conservation priorities is fundamentally influenced by values. Current conservation paradigms predominantly focus on “flagship” or charismatic species, biodiversity hotspots, rare or threatened taxa, and endangered habitats. Conservation strategy selection is invariably constrained by socio-political factors, economic feasibility, and operational constraints [16]. The ecological relationships between large umbrella species and their sympatric counterparts constitute a core mechanism for sustaining functional ecosystem stability [24]. As a flagship species in the forest ecosystems of Southeast Asia and southern China, the Asian elephant has been studied utilizing infrared camera technology to quantify temporal niche partitioning among associated species [25]. However, considerable gaps persist in systematically modeling the topological structure (e.g., association strength) within multispecies interaction networks. Selecting appropriate research designs and analytical pathways for these assessments presents considerable methodological challenges [26,27]. In 2023, the Nangunhe Asian Elephant Critical Habitat in Lincang, Yunnan, was designated on the inaugural List of Critical Terrestrial Wildlife Habitats (First Batch) in China. Within this reserve, extreme spatial compression confines the Asian elephant population to narrow valley corridors. This ecological insularization where populations become confined to isolated patches highlights the limitations. Species-centric management may temporarily relieve population decline; however, it fails to address the underlying systemic risks associated with collapsed interaction networks. Although protected area boundaries mitigate habitat loss, they simultaneously disrupt critical dispersal corridors required for species to perform their ecological functions [28]. Recent research on Asian elephants has predominantly emphasized single-species conservation, revealing critical gaps persist in the understanding of their ecological relationships with sympatric community assemblages. Studies leveraging interspecific interaction networks to inform conservation strategies remain critically underexplored. Temporal niche partitioning, primarily manifested through activity rhythms, constitutes a fundamental ecological dimension for the coexistence of sympatric species by facilitating resource differentiation [29]. Activity rhythms represent a key aspect of behavioral ecology, reflecting a species’ biological adaptation to environmental conditions. They reveal ecological strategies along the temporal axis and patterns of utilizing time as a resource [30]. The divergence in activity rhythms among species illustrates their strategies to minimize competition and optimize energy budgets, which is crucial for the survival and reproduction of sympatric species [29]. Therefore, investigating the activity patterns of co-occurring species is essential for elucidating the mechanisms that enable their coexistence [31].

To the best of our knowledge, this study pioneered reconstructing interaction networks in isolated elephant populations using infrared camera trapping and interspecific association modeling. We aimed to investigate the species composition, niche characteristics, competitive intensity, and species association patterns relative to community connectivity within isolated Asian elephant assemblages. The specific objectives were as follows: (1) elucidating species composition and ecological significance in Asian elephant communities; (2) determining niche characteristics (distributional breadth and spatial co-occurrence) among sympatric species; and (3) constructing spatial association networks for Asian elephants using interspecific association indices and community-wide connectivity metrics. Reintegrating species into their niche networks is essential for ensuring the resilient persistence of biodiversity. The findings of this study are anticipated to provide technical protocols for Asian elephant conservation and provide a theoretical basis for shifting conservation strategies from “species rescue” to “ecological relationship restoration.”

## 2. Materials and Methods

### 2.1. Study Area

This study was conducted in the NGHNNR (23°09′12″–23°40′08″ N, 98°57′32″–99°26′ E) in Yunnan Province, China, covering elevations of 510–2977 m a.s.l. and located merely 2.4 km from the China-Myanmar border. The reserve was established in 1980 and encompasses 508.87 km^2^ of the Nushan Range in the southern Hengduan Mountain System. The reserve straddles a transitional zone between the Shan Highlands of Myanmar and the Yunnan–Guizhou Plateau in China, encompassing the watersheds of the Lancang (Mekong) and Nu (Salween) Rivers. The region exhibits a subtropical monsoon climate, with a mean annual temperature of 9.6–22.8 °C and annual precipitation of 1280–2590 mm. It features distinct dry (November–April) and wet (May–October) seasons [32]. The reserve was primarily established to protect wildlife and their habitats. It features typical vertical vegetation zonation and supports an exceptionally diverse array of flora and fauna, ranking among global critical biodiversity hotspots. Its core conservation targets include the Asian elephant and tropical monsoon rainforest ecosystems. The reserve comprises 4 vertical vegetation zones, 9 major vegetation types, 16 vegetation subtypes, and 44 formations. It is rich in forest resources, which account for 98.77% of its total area, with woodland being the predominant type. The forest coverage rate is as high as 98.11% [33]. Notably, the β-clade of Asian elephants in China exists exclusively as an isolated subpopulation within Nangunhe [34]. Zoogeographically, the reserve lies within the Diannan Mountain Subregion (South China Region, Sino-Indian Subregion, Oriental Realm), delineating the northern limit of tropical fauna distribution and peripheral range of the Asian elephant. The reserve supports sympatric populations of protected herbivores, including northern red muntjac (*Muntiacus vaginalis* Zimmermann, 1780), sambar deer (*Rusa unicolor* Kerr, 1792), and Chinese serows (*Capricornis milneedwardsii* David, 1869), which share dietary niches with Asian elephants. The habitat for Asian elephants within NGHNNR is highly fragmented. This fragmentation is primarily anthropogenic, resulting from the expansion of agricultural land (e.g., coffee plantations) and settlements surrounding the reserve [6]. These human land uses have created a mosaic of forest patches within the reserve, particularly in the western core zone, isolating the elephant population and restricting their movement to narrow valley corridors.

### 2.2. Field Data Collection

**Figure 1:**
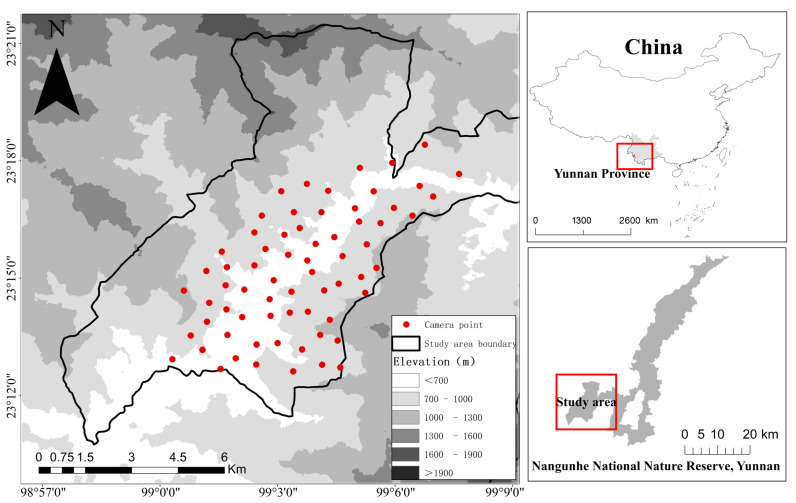
Schematic map of infrared camera trap deployment locations in the Nangunhe National Nature Reserve, Yunnan Province, China (Black polygon indicates the actual study area where camera traps were deployed).

Cameras were deployed within the known distribution range of Asian elephants, primarily covering the western core zone of the NGHNNR (approximate area: 36 km^2^). Before conducting the field surveys, we established a systematic 1-km^2^ grid system across the protected area using ArcGIS 10.8. A stratified sampling framework was implemented across the NGHNNR by integrating elephant movement patterns with environmental variables (topography, vegetation, and accessibility). This approach provided comprehensive coverage of the elephant distribution ranges, with one camera trap installed per 1-km^2^ grid cell for spatially explicit monitoring. We deployed 67 infrared camera traps (Ltl—6210 model, Leike Company, Guangzhou, China) across the reserve from June 2022 to June 2023 (Figure 1). We strategically deployed the camera traps with local expert guidance, maintaining ≥500 m spacing between sites. These precautions aimed to minimize the risk of accidental encounters between field personnel and elephas maximus during deployment and maintenance, which could potentially lead to conflict. Cameras were strategically positioned in core habitat types and areas with frequent elephant activity indicators (e.g., footprints, feces, and browsing marks) and mounted on tree trunks at a height of 50–150 cm to optimize frontal visibility and maximize animal detection rates. Each camera was configured to operate in hybrid photo-video mode, capturing three consecutive still images followed by a 30 s video recording upon motion detection, with a 1 min reactivation interval to minimize data redundancy. Quarterly maintenance was performed to ensure operational continuity and data retrieval.

We systematically cataloged and analyzed all photographic and video data. A capture-matrix methodology was used to perform pairwise comparisons of events, categorized as identical, non-identical, or indeterminate. Indeterminate classifications were documented along with their causes (e.g., poor resolution, suboptimal angles, and partial visibility). Images that failed to meet the quality thresholds for individual identification (e.g., blurring and overexposure) were excluded from the analyses. Species identification followed the standards outlined in A Guide to the Mammals of China [35] and A Field Guide to the Birds of China [36].

### 2.3. Data Analysis

The camera trap network served as a systematic array of sampling units across the landscape. The detection data (presence/absence, relative frequency) of a species across these units were used to infer its pattern of habitat utilization. The following indices analyze these patterns to quantify (1) how widely a species distributes itself across the available environmental conditions (distributional breadth), (2) how similarly two species distribute themselves (overlap in habitat use), and (3) the statistical associations between their occurrences.

#### 2.3.1. Relative Abundance of Species

The relative abundance index (*RAI*) refers to the proportion of individual counts or biomass of each species in a biological community relative to that of the total community [37]. In the present study, this was defined as the number of independent valid photographic captures per unit survey period (camera trap days) for a given species. To avoid duplicate records of species activities, the initial photograph of the same species captured within a 30 min period is considered an independent and valid detection [38].

Relative abundance reflects the ecological significance of different species within a community and serves as a critical component of population ecology [39]. Species with *RAI* values exceeding the mean were selected for subsequent analyses [37]. The *RAI* was calculated as follows:

(1) $$\mathrm{RAI}=\frac{n_{i}}{\sum_{i=1}^{S}n_{i}}\times 100$$

where *n_i_* is the number of independent photos of the *i* th species; and *S* is the total number of species.

#### 2.3.2. Distributional Breadth Across Sampling Sites

Niche width refers to the range or extent a species occupies within a multidimensional niche space, which is reflected in its ability to utilize diverse environmental resources and its tolerance to varying environmental conditions. To quantify distributional breadth, we employed two common indices calculated from species occurrence data across camera trap sites: the Levins index (*B_L_*) and an index derived from the Shannon-Wiener formula (*B_S_*). The latter applies the Shannon-Wiener formula to the proportional occurrence of a single species across sites, thereby measuring the evenness of its spatial distribution, which is interpreted as a component of its distributional breadth [40,41]. The *B_L_* and *B_S_* indices were calculated as follows:

(2) $$B_{L\;}=\;\frac{1}{\sum_{j=1}^{r}{\left(P_{\mathrm{ij}}\right)}^{2}}$$

(3) $$B_{s}=-\sum_{j=1}^{r}\left(P_{\mathrm{ij}}\ln P_{\mathrm{ij}}\right)$$

where *j* is the camera site; and *r* is the number of cameras. *Pij* represents the rate at which species i use camera site *j*.

#### 2.3.3. Overlap in Habitat Site Utilization

To estimate the degree of spatial association and potential habitat spatial co-occurrence between species pairs, we calculated the Pianka spatial co-occurrence index based on their co-occurrence patterns across camera trap sites. The spatial co-occurrence index [42] was selected using the following formula:

(4) $$O_{\mathrm{ik}}\;=\;\frac{\sum_{j=1}^{r}P_{\mathrm{ij}}P_{\mathrm{kj}}}{\sqrt{\left(\sum_{j=1}^{r}{P_{\mathrm{ij}}}^{2}\right)\left(\sum_{j=1}^{r}{P_{\mathrm{kj}}}^{2}\right)}}$$

where *O_ik_* is the spatial co-occurrence coefficient between species *i* and *k*; *P_ij_* and *P_kj_* denote the abundance of species *i* and *k* at the *j*-th camera site, respectively; and *r* is the total number of camera sites. The spatial co-occurrence index ranged from [0 to 1]. When *O_ik_* = 1, a complete overlap exists between species *i* and *k* at the *j*-th resource level. When *O_ik_* = 0, the two species do not share a common utilization of a particular resource.

#### 2.3.4. Overall Community Association

The integrity of faunal communities and species association networks serves as a critical indicator of ecosystem integrity and forms a foundation for community persistence and ecological functionality. The overall correlation among multiple species was assessed using the variance ratio (*V_R_*) method for community-wide association analysis [43]. Under the null hypothesis of independence, the expected value of the *V_R_* is 1. A *V_R_* > 1 indicates positive associations among species, whereas a *V_R_* < 1 suggests negative associations among species.

The significance of the *V_R_* was tested using the *W* statistic. If the W statistic fell within the 95% confidence interval of the chi-square distribution (χ^2^_0.95_ < *W* < χ^2^_0.05_), the overall species association was not statistically significant (*p* > 0.05). Conversely, if W fell outside this interval, the association was considered statistically significant (*p* < 0.05). The *V_R_* and *W* statistic were calculated as follows:

(5) $$V_{R}=\frac{1}{N}\sum_{j=1}^{N}\left(T_{j}-t\right)/\sum_{i=1}^{s}p_{i}\left(1-p_{i}\right)$$

(6) $$W={\;V}_{R}\times N$$

where *S* denotes the total number of species in the community; *p_i_* = *n_i_*/*N*; *N* is the total number of camera traps deployed; *n_i_* is the number of camera traps where species *i* was detected; *T_j_* is the total number of species recorded at camera site *j*; *t* is the mean number of species per camera trap across all surveyed sites.

#### 2.3.5. Calculation of Interspecific Association Indices

Interspecific associations quantify the spatial relationships between species within a community and reveal their distribution patterns of mutualism, competition, or independence. Based on the presence-absence data of species across camera trap locations, a 2 × 2 contingency table (Table 1) was constructed to analyze interspecific associations. Variables: *a*, number of sites where both species co-occurred; *b*, number of sites where only species A was present; *c*, number of sites where only species B was present; *d*, number of sites where neither species was detected.

The *Phi* correlation coefficient (*φ*) is a traditional approach for measuring interspecific associations [44], with φ values ranging from [−1, 1]. This formula is expressed as follows:

(7) $$\varphi \;=\;\frac{\mathrm{ad}-\mathrm{bc}}{\sqrt{\left(a+b\right)\left(a+c\right)\left(b+d\right)\left(c+d\right)\left(b+d\right)}}$$

In the equation, *φ* = 0 indicates that species are independent of each other with no association; *φ* < 0 signifies a negative association between species pairs; *φ* > 0 denotes a positive association between species pairs.

When analyzing small-sample 2 × 2 contingency tables, Yates’ Continuity Correction should be applied to minimize the discrepancy between discrete distributions and the continuous chi-square approximation, thereby ensuring the accuracy of statistical tests [45]. The corrected formula is expressed as follows:

(8) $$\chi_{\mathrm{Yates}}^{2}=\frac{{\left(\left|\mathrm{ad}-\mathrm{bc}\right|-0.5n\right)}^{2}n}{\left(a+b\right)\left(a+c\right)\left(b+d\right)\left(c+d\right)}$$

In this equation, *ad* − *bc* > 0 indicates a positive association between species; *ad* − *bc* < 0 signifies a negative association between species. The critical value table for the chi-squared (*χ*^2^) distribution indicates the following: *χ*^2^ < 3.841 (*p* > 0.05) signifies no significant association between species; 3.841 < *χ*^2^ < 6.635 (0.01 < *p* < 0.05) indicates a significant association between species; *χ*^2^ > 6.635 (*p* < 0.01) signifies an extremely significant association between species.

#### 2.3.6. Activity Rhythm Analysis

Diel activity rhythms of Asian elephants and sympatric species were analyzed using kernel density estimation [46]. In this approach, each camera-trap detection is treated as a random sample drawn from a continuous probability distribution of daily activity. The resulting kernel density curve represents the probability of detecting a species at any given time of day, with time plotted on the x-axis and the corresponding probability density (i.e., kernel density) on the y-axis. The area under each kernel density curve integrates to 1. Based on the timestamp data extracted from infrared camera photographs, we analyzed the degree of temporal overlap and segregation in activity patterns among species to explore the mechanisms underlying their coexistence [47]. The specific analytical steps were as follows:

(a) The range() function was used to convert the original clock times into decimal times (ranging from 0 to 1), which were subsequently transformed into radian units (decimal time × 2π). (b) Depending on the sample size of the smaller dataset within each species pair, an appropriate overlap coefficient (∆) was selected. The overlapEst() function from the overlap package 0.3.9 was then applied to calculate the daily activity rhythm overlap coefficient (∆, rounded to two decimal places) between paired species. (c) A smoothed bootstrap procedure with 1000 iterations was performed to generate 95% confidence intervals for the estimated overlap coefficients. (d) Daily activity curves for each species were plotted using the densityPlot() function from the overlap package. The smoothness of the curves was adjusted via the adjust parameter, where adjust = 0.8 was set for calculating ∆_1_ and adjust = 1 for ∆_4_. (e) Comparative activity plots for species pairs were generated using the overlapPlot() function. In this section, we incorporated the Chinese serow—a species with a foraging pattern similar to that of Asian elephants—into the analysis of daily activity rhythms.

All computations were conducted using the overlap package 0.3.9 [48] in R version 4.2.3.

#### 2.3.7. Analysis of Spatial Correlation Attributes of Species

In community ecosystems, species interact in many ways, each producing different effects. In some species interactions, only one party benefits from the interaction, whereas the other party experiences neither gain nor loss, exemplifying distinct commensal characteristics. From a spatial distribution perspective, beneficiary species typically aggregate around their associated species, which spatially attracts the beneficiary species to cluster, resulting in a unidirectional asymmetric association pattern between the species. When two species coexist within overlapping distribution ranges and exhibit mutual attraction, these interactions manifest as bidirectional or mutually asymmetric associations. Furthermore, in specific species interactions, neither party benefits from the association. In this case, the two species maintain independent spatial distributions with no attractive relationship between them. These interactions are characterized by symmetric associations. In this study, the Lambda coefficient was employed to test the associative characteristics between species [49], to determine the ecological relationships among species in the Asian elephant community.

Field data were collated using Microsoft Excel (2016). distributional breadth, spatial co-occurrence, and interspecific associations were analyzed and visualized using the spaa package 0.2.5 in R (version 4.2.3) and Origin 2021.

## 3. Results

### 3.1. Species Composition of the Captured Community

Across the 67 infrared camera traps deployed in the NGHNNR, 27,462 photographs and 8043 video clips were captured during the survey period. Following rigorous quality control and independent event screening, 1879 independent valid detections were identified for ecological analyses. A total of 44 animal species belonging to 28 families and 14 orders were identified (Table 2), encompassing mammals (Proboscidea, Artiodactyla, Primates, Rodentia, and Carnivora), birds (Galliformes, Accipitriformes, Passeriformes, Columbiformes, Piciformes, Coraciiformes, Bucerotiformes, and Strigiformes), and reptiles (Squamata). Eleven species with *RAI* values greater than the mean value (*RAI* = 0.109) were selected for subsequent interspecific association analyses.

### 3.2. Analysis of Niche Characteristics

#### 3.2.1. Niche Width

**Figure 2:**
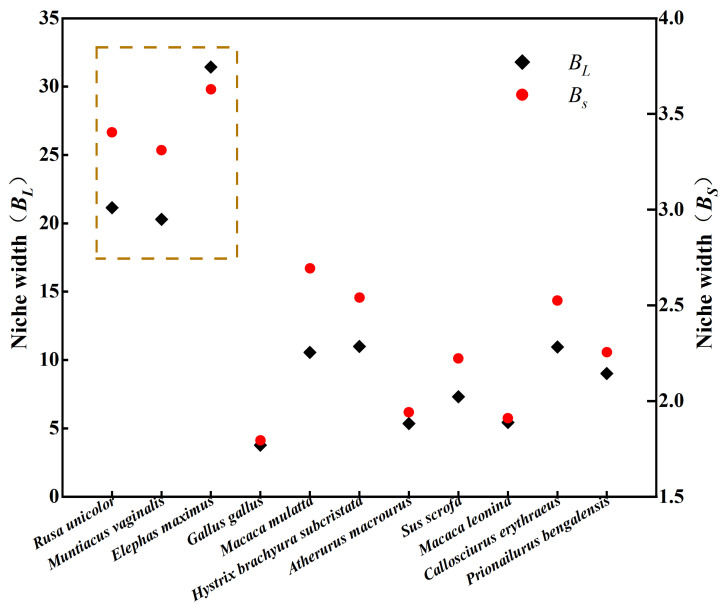
Variations in niche width (based on Levins and Shannon indices).

Based on Levins and Shannon indices, the Levins distributional breadth (*B_L_*) ranged from 3.77 to 31.43, whereas the Shannon distributional breadth (*B_S_*) ranged from 1.794 to 3.629. Within the community, the mean *BL* and *BS* were 12.38 and 2.56, respectively. Among all the species, sambar deer (*B_L_* = 21.14, *B_S_* = 3.4), northern red muntjac (*B_L_* = 20.29, *B_S_* = 3.31), and Asian elephants (*B_L_* = 31.43, *B_S_* = 3.63) exhibited the broadest niche widths, ranking the highest in both indices. Among the studied species, the Asian elephant exhibited the broadest distributional breadth, whereas the red junglefowl (*Gallus gallus* Linnaeus, 1758) showed the narrowest distributional breadth. Both distributional breadth metrics (Levins and Shannon indices) yielded congruent results, demonstrating consistent patterns across methodologies (Figure 2).

#### 3.2.2. Spatial Co-Occurrence

**Figure 3:**
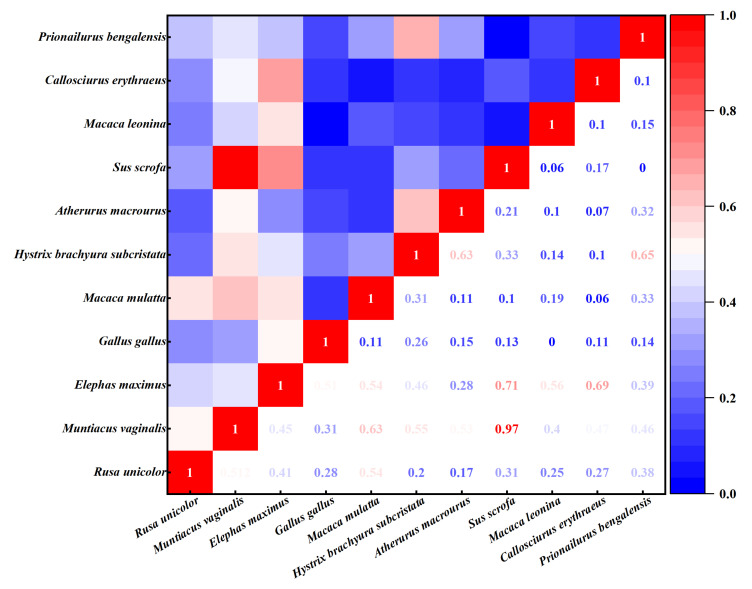
Interspecific spatial co-occurrence heatmap of the Asian elephant community.

The spatial co-occurrence among various species pairs within the community exhibited substantial variation. A total of 13 species pairs displayed spatial co-occurrence indices greater than 0.5, whereas 42 pairs showed indices below 0.5 (Figure 3). Overall, the interspecific spatial co-occurrence within the community remained relatively low, indicating substantial ecological differentiation among the major species, rational resource partitioning, and limited interspecific competition. Species with a relatively high spatial co-occurrence with the Asian elephant in the community included the wild boar (*Sus scrofa*, 0.71), red-bellied squirrel (*Callosciurus erythraeus*, 0.69), northern pig-tailed macaque (*Macaca leonina*, 0.56), macaque (*Macaca mulatta*, 0.54), and red junglefowl (0.51). Remarkably, the northern red muntjac and wild boar displayed the highest spatial co-occurrence (0.97) within the community, indicating either intense resource competition or shared habitat preferences.

#### 3.2.3. Daily Activity Rhythm

**Figure 4:**
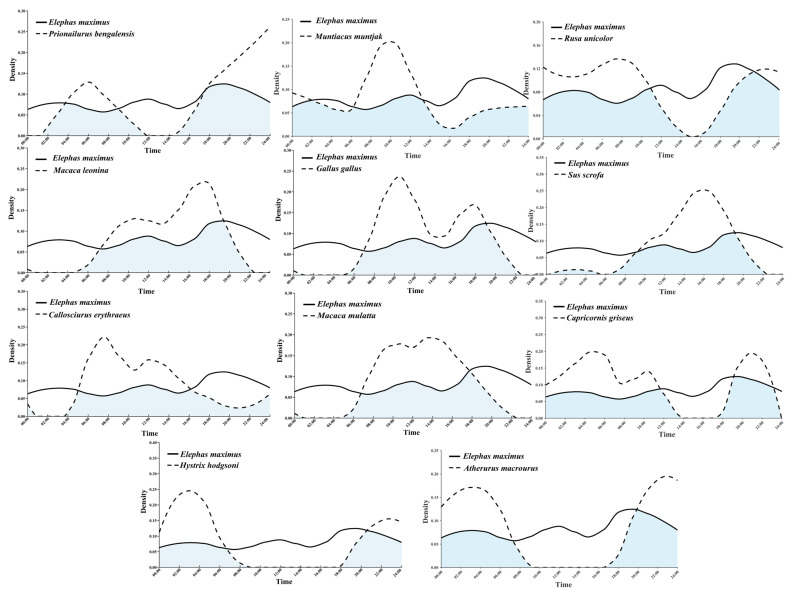
The overlapping diagram of the activity rhythms of Asian elephants and their sympatric species (blue represents the overlapping area).

Asian elephants exhibited a bimodal daily activity pattern, with peak activity concentrated during the dawn and dusk periods. The highest activity frequency was observed between 18:00 and 20:00 (Figure 4). The daily activity data for Asian elephants showed the smallest dispersion (lowest standard deviation), indicating a more stable activity rhythm. Comparisons of daily activity patterns between Asian elephants and the 11 sympatric species revealed varying degrees of overlap and divergence. The highest overlap occurred between Asian elephants and northern red muntjac (Δ = 0.59), while the lowest overlap was found between Asian elephants and Asiatic Brush-tailed Porcupine (*Atherurus macrourus*) (Δ = 0.37). No significant differences were detected between the daily activity rhythms of Asian elephants and any of the 11 other species (*p* > 0.05; see Supplementary Table S1).

### 3.3. Interspecific Association

#### 3.3.1. Overall Association

The overall association results of different species in the Asian elephant community (*V_R_* = 2.13 > 1) indicated that the overall association among species was positive. The statistic *W* = 142.95 (the range of *χ*^2^_0.95_ and *χ*^2^_0.05_ was 43.19–79.08), which was not within the *χ*^2^ value range, indicated a significant positive correlation among species within the community (*p* < 0.05).

#### 3.3.2. Asian Elephants and Their Spatially Associated Species Networks

**Figure 5:**
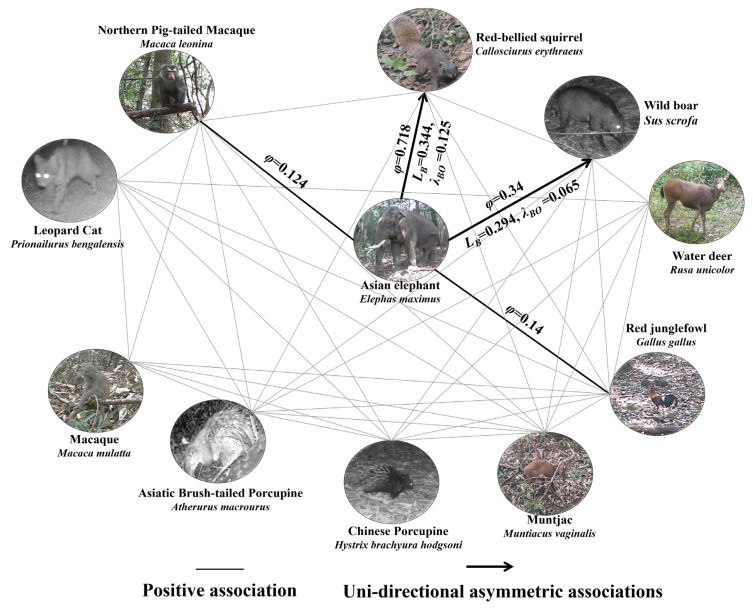
Interspecific association network diagram of Asian elephants.

Among the 67 camera trap locations, Asian elephants were detected at 50 locations, resulting in 304 independent detections. *Phi* correlation coefficient analysis revealed four species exhibiting positive associations with Asian elephants in the NGHNNR (Figure 5), including the red junglefowl (*φ* = 0.14, *χ*^2^ = 4.93, *p* < 0.05), wild boar (*φ* = 0.34, *χ*^2^ = 11.98, *p* < 0.01), northern porcupine macaque (*φ* = 0.124, *χ*^2^ = 4.37, *p* < 0.05), and red-bellied squirrel (*φ* = 0.718, *χ*^2^ = 25.32, *p* < 0.01).

In the Asian elephant spatial association network, symmetric spatial associations were observed between elephants, northern pig-tailed macaques, and red junglefowl, with no significant predictability between these species. Conversely, unidirectional asymmetric associations were identified between Asian elephants, red-bellied squirrels, and wild boars. The predictability rates of elephant presence for squirrel and boar distributions ranged from 0.125 to 0.344 and from 0.065 to 0.294, respectively, indicating that squirrels and boars were spatially attracted to elephant activity zones (Table 3).

## 4. Discussion

### 4.1. Spatial Distribution Pattern Differentiation and Resource Allocation Mechanism

The observed spatial distribution patterns and association networks provide a window into the community’s ecological structure. We interpret the broad distribution of Asian elephants as evidence of a broad spatial distribution pattern (generalist habitat use), while the strong asymmetric spatial dependence of wild boars on elephants suggests a commensal relationship facilitated by elephant engineering. Understanding the mechanisms underlying species coexistence in fragmented habitats is central to the field of community ecology [50]. We employed camera-trap data and niche analysis to quantify interspecific associations within an endangered Asian elephant community, elucidating spatial distribution patterns and ecological linkages with sympatric species. The niche-breadth-range-size hypothesis posits that generalist species possess broad niches that enable them to tolerate diverse environmental conditions and exploit multiple resources [51].

Niche analysis confirmed that Asian elephants exhibited the broadest distributional breadth. This finding aligns with their migratory behavior and dietary plasticity [4]. In contrast, specialists, such as the red junglefowl, depend on specific vegetation types, reflecting resource partitioning within the community. Wild boar and northern red muntjac exhibited the highest spatial co-occurrence (0.97). While this pattern could be interpreted as potential competition for shared habitat or resources, their divergent spatial associations with elephants—positive for boars and neutral for northern red muntjac—suggest a more complex interaction. We hypothesize that Asian elephants, through their well-documented role as ecosystem engineers [9], may indirectly modify the competitive landscape. For instance, elephant foraging and movement create microhabitat heterogeneity (e.g., forest gaps, waterholes) [9]. These modified sites could provide alternative resources for species like wild boar (e.g., rooting in disturbed soil), potentially reducing direct competition with muntjac at finer spatial scales. This aligns with the concept that keystone species can promote community structure not only through top-down regulation but also via habitat facilitation [22]. Thus, our observed spatial network is consistent with Asian elephants playing a critical role in shaping resource availability and species interactions in this isolated habitat, though the precise mechanistic pathways require further investigation.

### 4.2. Temporal Niche Partitioning and Multi-Dimensional Coexistence

Our analysis of daily activity rhythms adds a critical temporal dimension to understanding community coexistence. The stable bimodal pattern of Asian elephants, peaking at crepuscular hours, aligns with strategies for thermoregulation and possibly avoidance of human disturbance in this fragmented landscape. The lowest temporal overlap with the Asiatic Brush-tailed Porcupine (Δ = 0.37) suggests temporal segregation may facilitate coexistence with this potentially competing nocturnal rodent. Conversely, the highest overlap with the northern red muntjac (Δ = 0.59). Given their high spatial co-occurrence (*O_ik_* = 0.97), this implies that these two herbivores may coexist in the same spaces at similar times, potentially relying on subtle differences in microhabitat use or dietary preferences not captured here—a hypothesis supporting the idea of “niche packing” in stable communities. The absence of significant temporal avoidance across all species pairs suggests that time may not be the primary axis for competitive release in this system. Instead, the integration of spatial associations, habitat use patterns, and temporal activity data depicts a community where Asian elephants, as the keystone engineer, shape a landscape of opportunity. Sympatric species appear to partition this landscape through a combination of mechanisms: some (e.g., wild boar) track elephant activity spatially, others (e.g., porcupine) may separate temporally, while many (e.g., muntjac) share both time and space but likely utilize different subsets of resources within elephant-modified habitats. This multi-faceted perspective underscores the complexity of interaction networks and reinforces the necessity of moving beyond single-dimensional analyses in conservation planning.

### 4.3. Keystone Species Status of Asian Elephants and Ecological Network Construction

The Asian elephant serves as a quintessential ecosystem engineer, shaping communities and maintaining ecosystem functions through diverse mechanisms. As a flagship species, its activities greatly influence the distribution patterns of numerous co-occurring species within its range. Red-bellied squirrels and wild boars exhibited unidirectional asymmetric associations with elephants, suggesting a potential commensal relationship through their spatial association with elephant activity zones. This commensalism likely stems from the habitat modification effects of Asian elephants. By feeding on tree bark, elephant herds create forest gaps and deposits of fallen trees, which serve as foraging platforms and nesting materials for arboreal species, such as red-bellied squirrels. Additionally, the temporary water holes they dig function as critical water sources for small- to medium-sized animals, such as wild boars and red junglefowl, particularly during the dry season (November–April), while also offering sites for foraging and shelter [9]. For example, the aggregative response of wild boars to areas of elephant activity may be linked to their utilization of plant roots and insects exposed in elephant-tilled soils [2]. This passive symbiotic pattern was evident in our study through the spatial tracking of elephant ranges by wild boars and red-bellied squirrels. This finding supports the core role of Asian elephants as habitat providers. Furthermore, the omnivorous diet and migratory behavior of Asian elephants position them as key seed dispersers in tropical rainforest ecosystems [4]. Transporting seeds across landscapes maintains plant diversity and indirectly provides food resources for rodent and primate species dependent on these plants. Notably, the symmetric associations between Asian elephants, northern pig-tailed macaques, and red junglefowl reflect the absence of clear resource dependence or competition, illustrating diverse coexistence strategies within the community. As sympatric herbivores with potentially overlapping dietary niches, the low detection rate of the Chinese serow in our study area may reflect spatial avoidance of Asian elephant activity zones. This in itself represents an important ecological relationship, demonstrating the indirect competitive effects that elephant activities may generate. These findings reinforce the hypothesis that Asian elephants promote biodiversity in tropical forests via habitat modification [52].

Community structure in fragmented habitats is influenced by both environmental filters and species interactions. In complex species networks, interspecific associations mediate the balance between these two relationships [53]. Our finding of significant positive overall associations among the detected species suggests that the community within the elephant’s range may be stabilized by a preponderance of neutral or facilitative relationships [22]. This is evidenced in the spatial association network centered on Asian elephants, which is predominantly composed of herbivores such as sambar deer and northern red muntjac. Our camera data show that these species frequently co-occur with elephants in the same areas. This co-occurrence pattern is consistent with the well-documented role of elephants as ecosystem engineers whose foraging creates forest gaps and trampled clearings, which are reported to provide fresh vegetation regrowth for other herbivores [9,25]. Similarly, primates like northern pig-tailed macaques exhibited neutral associations with elephants, a pattern that could be consistent with their utilization of different strata or resources in elephant-modified habitats without direct competition. Conversely, the scarcity of medium-to-large carnivores in the observed network may reflect the combined effects of habitat fragmentation on apex predators and a potential spatial avoidance of intensive elephant activity zones. Together, these observed spatial patterns reinforce the concept of the Asian elephant as a “keystone modifier” whose presence is a major structuring force for the resident community, although the specific mechanisms of facilitation warrant further investigation.

With no natural predators (excluding humans), the size and landscape engineering capabilities of Asian elephants play a keystone role in community stability. However, excessive reliance on the “artificial microhabitats” created by Asian elephants poses risks: small mammals, such as red-bellied squirrels, that overly depend on elephant-provided food resources (e.g., insects and exposed roots in tilled soils) may lose their foraging independence. This dependency can result in high mortality rates during seasonal resource shortages [28]. Additionally, specific species, such as the Chinese serow, which exhibits weak negative associations with elephants, can be subtly displaced to suboptimal habitats. This highlights the trade-offs between facilitation and indirect competition in elephant-dominated communities. The Lambda coefficient offers a novel approach for quantifying asymmetric interspecific associations. By quantifying the asymmetry of associations through the upper and lower bounds of the predictability rates, we successfully identified the unidirectional spatial dependence of wild boars and red-bellied squirrels on Asian elephants [50]. In contrast to the traditional Phi coefficient, which only reveals the direction and strength of associations, the Lambda coefficient is better suited for dissecting commensal relationships between “ecosystem engineers and associate species,” thereby providing a new dimension for understanding functional dependencies at the community level.

### 4.4. Conservation of Asian Elephants and Ecological Risks Associated with Isolated Populations

As a flagship species in tropical forest ecosystems, the Asian elephant enhances biodiversity and sustains vital ecosystem services. China has enacted substantial conservation measures for Asian elephants, establishing protected areas, such as the Xishuangbanna National Nature Reserve and NGHNNR, both of which prioritize elephant conservation. In Xishuangbanna, the Asian Elephant Rescue and Breeding Center was established to rehabilitate injured individuals, while road infrastructure development incorporated ecological corridors to accommodate elephant migration and preserve habitat connectivity [54]. These initiatives have fostered favorable habitat conditions for the resident Asian elephant population in Xishuangbanna, demonstrating the effective integration of conservation and landscape management.

Suitable habitats for Asian elephants are limited to the NGHNNR. Surrounding villagers have cleared forests to plant cash crops, such as coffee, thereby obstructing elephant migration to Myanmar and resulting in the current pattern of ecological insularization [55]. Our research team has conducted extensive conservation work on Asian elephants in China and found that topographical factors are key determinants of their distribution, whereas vegetation coverage is critical for their habitat use. Currently, the habitats in the western core zone of the reserve are insufficient. Although suitable habitats exist in other areas of the western core zone, a hydropower station at the east–west junction prevents elephant dispersal to the east. Additionally, settlements to the west, south, and north further limit their range. These factors confine Asian elephants to the western core area. The highly dependent associations identified in our network analysis indicate that habitat fragmentation of elephant populations may threaten the associated species through cascading effects [28]. Human–elephant conflict represents the greatest challenge for elephant conservation and management in China; however, no cases of retaliatory killings due to crop raiding have occurred. Faced with an elevated risk of extinction, the NGHNNR Asian elephant population necessitates the involvement of local residents in conservation efforts to reduce cash crop cultivation, identify potential ecological corridors, and enhance habitat connectivity. Introducing new Asian elephant individuals can also mitigate the loss of genetic diversity. These measures demand proactive engagement and support from both governmental entities and local communities [6]. Future conservation strategies should prioritize the dual goals of “species-network” protection and connectivity restoration to maintain the ecosystem engineering functions of elephants and avoid secondary risks from network collapse [16].

### 4.5. Limitations and Future Directions

Our study focused on species with higher detection rates (*RAI*) for network analysis, which may underrepresent ecologically important but rare or elusive species (e.g., *Capricornis milneedwardsii*, *Ursus thibetanus*). Future studies should integrate species’ conservation status, functional traits, and occupancy estimates to construct more comprehensive interaction networks for conservation planning. Our measurement of distributional breadth is based on spatial occurrence, serving as a proxy for the breadth of environmental conditions a species utilizes. While this effectively captures the realized spatial dimension of the niche, future studies incorporating direct data on diet (e.g., DNA metabarcoding of feces) and microhabitat use would allow for a more granular, multi-dimensional niche analysis.

## 5. Conclusions

This study conducted an investigation on the isolated Asian elephant population distribution in the NGHNNR in China, and identified a total of 44 species that are distributed in the same area. Asian elephants have the widest spatial distribution pattern, which can influence the activity patterns of species in the same habitat. This study indicates that Asian elephants in the NGHNNR exhibit broad distributional breadth and spatial associations with sympatric species, including wild boars and red-bellied squirrels, thereby illustrating a unidirectional commensal relationship. Overall, the positive associations within the community reflect a stable mutualistic interaction network that is critical for ecosystem stability. Our findings advocate for a paradigm shift in conservation: from single-species protection to ‘interaction network conservation’. Specifically, we recommend the following: (1) Using association networks as a monitoring tool: Species like wild boar and red-bellied squirrel, which show strong spatial reliance on elephants, can serve as indicators for assessing the quality and connectivity of elephant habitats. (2) Designing corridor with network in mind: Habitat restoration and corridor planning should aim to preserve not only elephant movement paths but also the microhabitat features (e.g., water holes, forest gaps) that support their associated species community. This integrated approach is crucial for ensuring the long-term viability of both the isolated Asian elephant population and the entire ecological network it supports.

## Figures and Tables

**Table 1:** 2 × 2 linked species distribution table.

Species A	Species B		
	Presence	Absence	Total
Presence	*a*	*b*	*a *+* b*
Absence	*c*	*d*	*c *+* d*
Total	*a *+* c*	*b *+* d*	*N *=* a *+* b *+* c *+* d*

**Table 2:** Species composition and relative abundance of the Asian elephant community in the Nangunhe National Nature Reserve.

Orders	Family	Species	Number of Individual Photographs	Relative Abundance Index	Number of Camera Sites Detected (Occupancy)
**Mammalia**					
Proboscidea	Elephantidae	***Elephas maximus* Linnaeus, 1758**	307	74.6 *	50
Artiodactyla	Cervidae	***Rusa unicolor* Kerr, 1792**	731	91.0 *	61
		***Muntiacus vaginalis* Zimmermann, 1780**	381	65.7 *	44
	Suidae	***Sus scrofa* Linnaeus, 1758**	32	17.9 *	12
	Bovidae	*Capricornis milneedwardsii* Bechstein, 1799	21	10.4	7
Primates	Cercopithecidae	***Macaca mulatta* Zimmermann, 1780**	80	32.8 *	**22**
		***Macaca leonina* Blyth, 1863**	21	13.4 *	**9**
		*Trachypithecus crepusculus* Elliot, 1909	1	1.5	1
Rodentia	Hystricidae	***Atherurus macrourus* Linnaeus, 1758**	33	14.9 *	**10**
		***Hystrix brachyura subcristata* Swinhoe, 1870**	58	23.9 *	**16**
	Sciuridae	***Callosciurus erythraeus* Pallas, 1778**	19	20.9 *	**14**
Carnivora	Felidae	***Prionailurus bengalensis* Kerr, 1792**	12	14.9 *	**10**
	Mustelidae	*Melogale moschata* Gray, 1831	5	4.5	3
		*Martes flavigula* Boddaert, 1785	3	4.5	1
	Viverridae	*Viverricula indica* Geoffroy, 1803	5	3.0	2
		*Paradoxurus hermaphroditus* Pallas, 1777	8	4.5	3
		*Paguma larvata* Smith, 1827	1	1.5	1
	Ursidae	*Ursus thibetanus* G. Baron Cuvier, 1823	2	1.5	1
**Aves**					
Galliformes	Phasianidae	***Gallus gallus* Linnaeus, 1758**	101	18.0 *	**12**
		*Lophura nycthemera* Linnaeus, 1758	11	9.0	6
		*Arborophila atrogularis* Blyth, 1850	1	1.5	1
Accipitriformes	Accipitridae	*Pernis ptilorhynchus* Temminck, 1821	1	1.5	1
		*Spilornis cheela* Latham, 1790	3	3.0	2
Passeriformes	Sittidae	*Sitta nagaensis* Godwin-Austen, 1874	1	1.5	1
		*Sitta frontalis* Swainson, 1820	1	1.5	1
	Turdidae	*Turdus dissimilis* Blyth, 1847	13	10.4	1
	Phylloscopidae	*Phylloscopus tephrocephalus* Anderso, 1871	1	1.5	1
	Pellorneidae	*Pellorneum ruficeps* Swainson, 1832	3	4.5	3
		*Schoeniparus dubius* Hume, 1874	1	1.5	1
	Timaliidae	*Pomatorhinus schisticeps* Hodgson, 1836	1	1.5	1
	Leiothrichidae	*Leiothrix argentauris* Hodgson, 1837	1	1.5	1
	Muscicapidae	*Copsychus malabaricus* Scopoli, 1786	1	1.5	1
		*Copsychus saularis* Linnaeus, 1758	2	1.5	1
		*Myophonus caeruleus* Scopoli, 1786	3	3.0	2
	Pycnonotidae	*Alophoixus flaveolus* Gould, 1836	1	1.5	1
	Alcippeidae	*Alcippe fratercula* Rippon, 1900	1	1.5	1
Columbiformes	Columbidae	*Streptopelia decaocto* Frivaldszky, 1838	1	1.5	1
		*Chalcophaps indica* Linnaeus, 1758	4	6.0	4
		*Macropygia unchall* Wagler, 1827	2	1.5	1
Piciformes	Picidae	*Chrysophlegma flavinucha* Gould, 1834	1	1.5	1
Coraciiformes	Meropidae	*Nyctyornis athertoni* Jardine & Selby, 1828	1	1.5	1
Bucerotiformes	Bucerotidae	*Anthracoceros albirostris* Shaw & Nodder, 1807	1	1.5	1
Strigiformes	Strigidae	*Glaucidium cuculoides* Vigors, 1831	1	1.5	1
**Reptilia**					
Squamata	Varanidae	*Varanus salvator* Laurenti, 1768	1	1.5	1

Note: * indicates species whose relative abundance index was greater than the mean relative abundance index (*RAI* = 10.9).

**Table 3:** Lambda coefficient tests between Asian elephants and their associated species.

Predicting Species	Predicted Species	*L_B_* (Upper)	*λ_BO_* (Lower)	*p*
Asian elephant	Red Junglefowl	0.162	−0.093	*p* > 0.05
Asian elephant	Wild boar	0.294	0.065	*p* ≤ 0.05
Asian elephant	Northern pig-tailed macaque	0.471	−0.21	*p* > 0.05
Asian elephant	Red-bellied squirrel	0.344	0.125	*p* ≤ 0.05
Red Junglefowl	Asian elephant	0.041	−0.017	*p* > 0.05
Wild boar	Asian elephant	0.060	−0.417	*p* > 0.05
Northern pig-tailed macaque	Asian elephant	0.109	−0.890	*p* > 0.05
Red-bellied squirrel	Asian elephant	0.091	−0.214	*p* > 0.05

Note: *L_B_* denotes the upper limit of the prediction rate, while *λ_BO_* denotes the lower limit of the prediction rate.

## Data Availability

The original contributions presented in this study are included in the article/Supplementary Materials. Further inquiries can be directed to the corresponding author (Dehuai Meng).

## Supplementary Materials

The following supporting information can be downloaded at: https://www.mdpi.com/article/10.3390/ani16020351/s1, Table S1: Differences in daily activity rhythms among Asian elephants and their sympatric species.
