# Supplementary material for: Spatial Associations and Co-Occurrence Networks of Sympatric Species in an Asian Elephant Community
Source: Animals (Basel). 2026 Jan 22;16(2):351. doi: 10.3390/ani16020351 (PMC12837749; doi:10.3390/ani16020351)
Supplement: Supplementary file 1 [file animals-16-00351-s001.zip › Table S1.pdf]

Table S1: Differences in daily activity rhythms among Asian elephants and their sympatric species

**Species Combination (*Elephas maximus*)**	**The Overlap Degree of Daily Activity Rhythms Δ**	**Confidence Interval (95%)**	**Significance *p***
Prionailurus bengalensis	0.4	0.16–0.68	>0.05
Macaca leonina	0.39	0.15–0.68	>0.05
Callosciurus erythraeus	0.43	0.18–0.64	>0.05
Muntiacus vaginalis	0.59	0.29–0.80	>0.05
Gallus gallus	0.38	0.17–0.62	>0.05
Macaca mulatta	0.53	0.29–0.72	>0.05
Rusa unicolor	0.55	0.25–0.80	>0.05
Sus scrofa	0.38	0.19–0.60	>0.05
Hystrix brachyura hodgsoni	0.47	0.27–0.73	>0.05
Capricornis milneedwardsii	0.43	0.21–0.59	>0.05
Atherurus macrourus	0.37	0.16–0.58	>0.05
